# Imatinib could be a new strategy for pulmonary hypertension caused by pulmonary tumor thrombotic microangiopathy in metastatic breast cancer

**DOI:** 10.1186/s40064-016-3280-4

**Published:** 2016-09-15

**Authors:** Ippei Fukada, Kazuhiro Araki, Kokoro Kobayashi, Tomoko Shibayama, Masaru Hatano, Shunji Takahashi, Takuji Iwase, Shinji Ohno, Yoshinori Ito

**Affiliations:** 1 Breast Medical Oncology, Breast Oncology Center, The Cancer Institute Hospital of the Japanese Foundation for Cancer Research, 3-8-31, Ariake, Koto-ku, Tokyo, 135-8550 Japan; 2 Cardiovascular Medicine, The University of Tokyo Hospital, Tokyo, Japan; 3 Medical Oncology, The Cancer Institute Hospital of the Japanese Foundation for Cancer Research, Tokyo, Japan; 4 Breast Surgical Oncology, Breast Oncology Center, The Cancer Institute Hospital of the Japanese Foundation for Cancer Research, Tokyo, Japan; 5 Breast Oncology Center, The Cancer Institute Hospital of the Japanese Foundation for Cancer Research, Tokyo, Japan

**Keywords:** Pulmonary tumor thrombotic microangiopathy, Imatinib, Metastatic breast cancer, Pulmonary hypertension

## Abstract

**Introduction:**

Pulmonary tumor thrombotic microangiopathy (PTTM) is rare, cancer-related pulmonary complication leading to hypoxia, pulmonary hypertension, and heart failure. The standard treatment for PTTM is not established. However, imatinib, a tyrosine kinase inhibitor of the PDGF receptor, may cause regression of pulmonary hypertension and pulmonary artery remodeling in PTTM.

**Case descriptions:**

We report two cases of PTTM who received an anti-PDGF agent of imatinib for PTTM developed during chemotherapy for metastatic breast cancer. Case 1: 61-year-old woman who underwent resection of the left breast and axillary lymph node dissection and received adjuvant chemotherapy (CAF followed by docetaxel), then endocrine therapy for 5 years. Twelve years after surgery, multiple bone and mediastinal lymph node metastases occurred. She was under treatment with eribulin for one year but admitted because of rapid progressing dyspnea. Case 2: 45-year-old woman with metastatic breast cancer in multiple bones was under treatment for 5 years. Receiving capecitabine, she suffered from dyspnea for 2 months, she was admitted to our hospital with diagnosis of severe hypoxia. In both cases, the wedged pulmonary arterial blood cell sampling revealed cytologically malignant cells which confirmed the diagnosis of PTTM. They were treated with imatinib, which alleviated pulmonary hypertension. However, they died due to progression of metastatic breast cancer.

**Discussion and Evaluation:**

Single use of imatinib did not showed sufficient efficacy. It is necessary to conduct a well-designed clinical trial using chemotherapies combined with imatinib for PTTM.

**Conclusions:**

Imatinib, which alleviated pulmonary hypertension, could be a new strategy for pulmonary tumor thrombotic microangiopathy in patient with metastatic breast cancer.

## Background

Pulmonary tumor thrombotic microangiopathy (PTTM) is very rare cancer-related complication that causes pulmonary hypertension, heart failure and hypoxia. Although the standard treatment for PTTM is not established, imatinib, a tyrosine kinase inhibitor of the PDGF receptor, may cause regression of pulmonary hypertension and pulmonary artery remodeling in PTTM. We report two cases of PTTM in patients with metastatic breast cancer who receiving imatinib for pulmonary hypertension caused by PTTM.

## Case presentation

### Case1

**Fig. 1:**
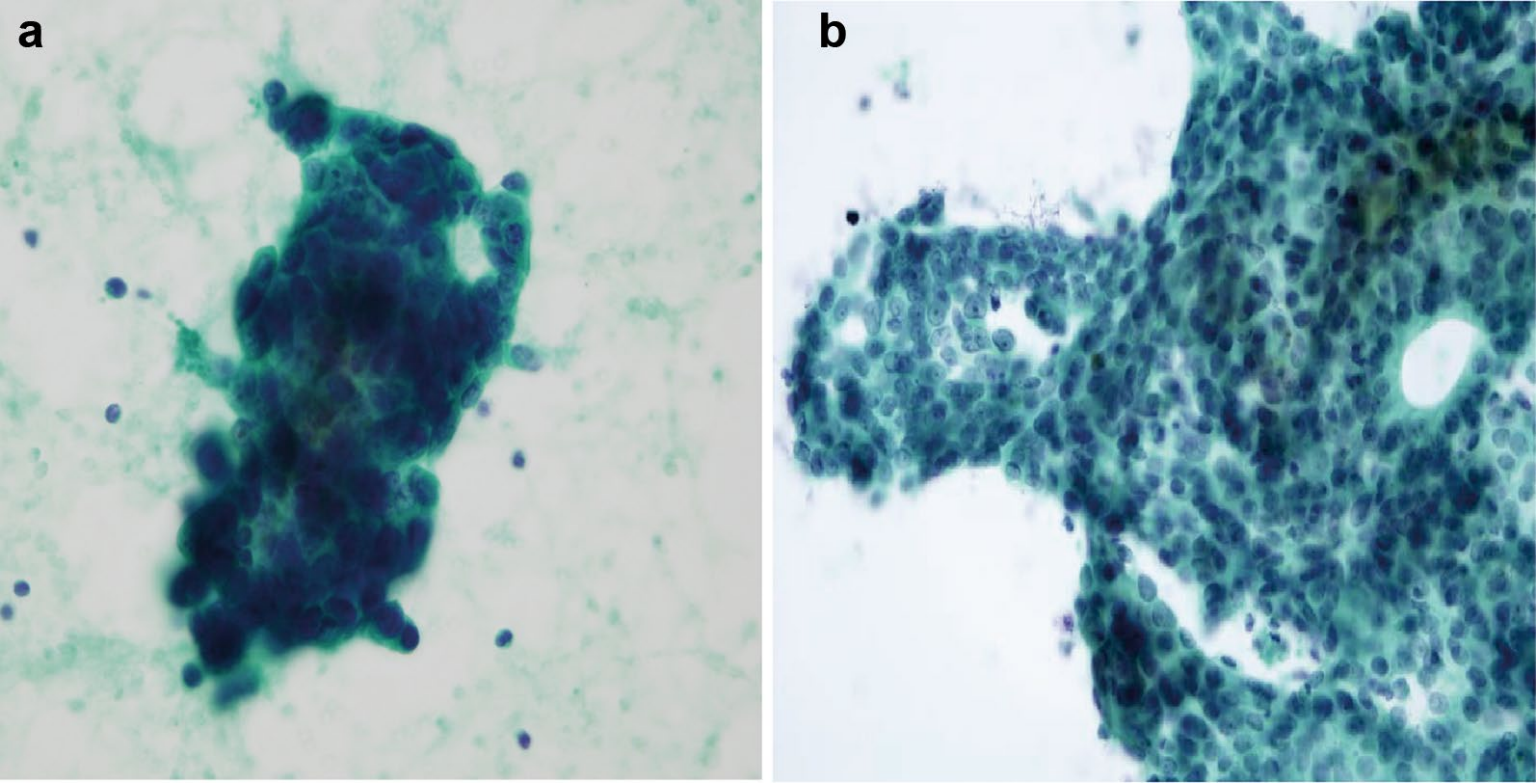
Cytology from pulmonary artery (**a** case 1, **b** case 2). The cytology of the blood in the pulmonary artery revealed adenocarcinoma cells (Papanicolaou stain)

The patient was a 61-year-old woman who had undergone resection of the left breast and axillary lymph node dissection. She received adjuvant chemotherapy (CAF; cyclophosphamide 500 mg/m^2^, doxorubicin 50 mg/m^2^, fluoropyrimidine 500 mg/m^2^ followed by docetaxel, then tamoxifen followed by anastrozole for a total of 5 years. Twelve years after surgery, multiple bone and mediastinal lymph node metastases were detected. She was under treatment with eribulin for 1 year but admitted because of rapid progressing dyspnea. Although the enhanced computed tomography showed no pulmonary embolism, ventilation-perfusion scintigraphy demonstrated multiple small peripheral perfusion defects in both lungs. A transthoracic echocardiogram showed severe pulmonary hypertension with estimated right ventricular systolic pressure of 76 mmHg. She was transferred to the Department of Cardiovascular Medicine, the wedged pulmonary arterial blood cell sampling showed clusters of malignant cells with high nuclear/cytoplasm ratio, focal glandular structures which confirmed the diagnosis of PTTM (Fig. 1a). Pulmonary arterial pressure (PAP) was measured at 93/39(60) mmHg, the cardiac index (CI) was 1.63 L/min/m^2^ and pulmonary vascular resistance elevated to 1947 dyne·s/cm^5^. Nine days after administering the imatinib (200 mg/day), the PAP was reduced to 87/30(50) mmHg, and the CI was improved to 2.83 L/min/m^2^. We increased the dose of imatinib to 400 mg, then the CI was improved to 2.97 L/min/m^2^ though the PAP was slightly elevated to 95/44(56) mmHg (Table 1). The patient died due to progression of breast cancer itself 54 days after her initial admission. In autopsy, an embolus of tumor cells was noted in the pulmonary artery and the lumen of the pulmonary artery was severely narrowed. Tumor cells were immunohistochemically positive for PDGF-B.

**Table 1:** The result of catheterization study in case1 and case2

	Case 1			Case 2	
	Day 1	Day 7	Day 22	Day 1	Day 7
Medication	None	Imatinib 200 mg	Imatinib 400 mg	None	Imatinib 200 mg
PAP (mmHg)	93/39 (60)	87/30 (50)	95/40 (56)	47/18 (29)	45/22 (30)
CI (L/min/m^2^)	1.63	2.83	2.97	2.98	3.65
PVR (dyne·s/cm^2^)	1947	914	1037	490	410

### Case2

A 45-year-old woman with metastatic breast cancer in multiple bones. The core needle biopsy from primary breast tumor revealed that invasive ductal carcinoma with nuclear grade1, ER+ , PgR+ , HER2:0. She was under treatment for 5 years (Tamoxifen, FAC therapy; Cyclophosphomide 500 mg/m^2^, Doxorubicin 50 mg/m^2^, Fluorouracil 500 mg/m^2^ for 7 months, triweekly docetaxel for 11 months, anastrozole and leuprorelin for 21 months, and capecitabine for 8 months with zoledronic acid. After she suffered from dyspnea for 2 months, she was admitted to our hospital with diagnosis of severe hypoxia. Although the enhanced computed tomography did not show pulmonary embolism, the scintigraphy demonstrated patchy flow reduction in both lungs. The test of blood coagulation suggested the micro-thromboembolism. Hypoxia rapidly progressed even after anticoagulation therapy. On the 21st day after admission, she was transferred to the Department of Cardiovascular Medicine for a more precise diagnosis and intensive treatment. Wedged pulmonary arterial blood cell sampling revealed cytologically malignant cells which confirmed the diagnosis of PTTM (Fig. 1b). The serum of the patient exhibited a high level of platelet-derived growth factor (PDGF), which produced by tumor cells, would promote vascular remodeling. Pulmonary arterial pressure (PAP) was measured at 47/18(27) mmHg, and the cardiac index (CI) was 2.98 L/min/m^2^. Pulmonary vascular resistance elevated to 490 dyne·s/cm^5^. Imatinib (200 mg/day) was administered as a clinical trial immediately. Seven days administering imatinib, the PAP was still 45/22(30) mmHg, but the CI was improved to 3.65 L/min/m^2^ and pulmonary vascular resistance reduced to 400 dyne·s/cm^5^ (Table 1). Although we increased the dose of imatinib to 400 mg, she died of respiratory insufficiency in 24 days from her initial admission.

## Discussion

We demonstrated that imatinib could alleviated pulmonary hypertension in two patient with PTTM. PTTM is one of the very rare cancer-related pulmonary complications, leading to pulmonary hypertension, heart failure and hypoxia. Von Herbay et al. reported 21 cases of PTTM in 630 carcinoma autopsies, the incidence of PTTM was 3.3 % (von Herbay et al. 1990). In their report, all 21 cases had carcinoma with distant metastases and 19 cases had adenocarcinomas of various organs. Although the stomach cancer was the most common, there was two cases of breast cancer. The other previous reports by Okubo et al. showed six cases of PTTM in 37 gastric carcinoma autopsies (Okubo et al. 2011). The mechanism of PTTM is still unclear. However, the morphometric and immunohistochemical analyses have been developed recently. There are two hypotheses for the development of pulmonary hypertension and right-heart failure, the dysregulation of signaling pathways which respond to the presence of an embolic cell or other intravascular insult, causes vascular remodeling and the other hypothesis proposes that tumor emboli occlude the pulmonary arterial bed and increase pulmonary vascular resistance (Roberts et al. 2003). Von Herbay et al. revealed that tumor cells invaded the pulmonary vascular system and occluded the small arteries and arterioles that activate coagulation systems, releasing inflammatory mediators and growth factors, which induced both local activation of coagulation and fibrocellular intimal proliferation (von Herbay et al. 1990; Sakashita et al. 2007). Okubo et al. also reported the morphometric analysis of pulmonary arteries and suggested that pulmonary arterial remodeling induced by carcinoma cell adhesion onto the endothelium affected the status of pulmonary hypertension. Some previous studies revealed that cancer cells produced the molecules that cause PTTM, which contains the tissue factor (Okubo et al. 2011), the vascular endothelial growth factor (Sakashita et al. 2007; Chinen et al. 2010; Takahashi et al. 2009; Yokomine et al. 2010) and osteopontin (Takahashi et al. 2009; Denhardt et al. 2001). The platelet-derived growth factor (PDGF) and PDGF receptor (PDGFR) is also important molecular agents in development of pulmonary hypertension in patients with PTTM (Abe et al. 2013). von Herbay et al. (1990) reported that attachment of tumor cell emboli might damage endothelial cells, releasing PDGF (von Herbay et al. 1990). Yokomine et al. reported an autopsied case of PTTM with gastric carcinoma that expressed the PDGF and PDGFR in cancer cells. They also revealed that the overexpression of PDGF was detected in alveolar macrophages and PDGFR in intimal mesenchymal cells in the pulmonary arterial wall, which suggested the contribution of the activated alveolar macrophages to the onset of PTTM (Yokomine et al. 2010). The standard treatment for PTTM has not been established. However, imatinib, a tyrosine kinase inhibitor of the PDGF receptor, may cause regression of pulmonary hypertension and pulmonary artery remodeling in PTTM (Ogawa et al. 2013). In our cases, imatinib was administered as a clinical trial, approved by the Institutional Review Board of the University of Tokyo Hospital. Although imatinib alleviated pulmonary hypertension and the cardiac index temporarily improved after administration of imatinib in both cases, they died due to progression of metastatic breast cancer. Single use of imatinib did not showed sufficient efficacy to suppress the disease progression of breast cancer itself. Therefore, it is necessary to establish a new strategy for PTTM in metastatic breast cancer, using the combination of imatinib and the other chemotherapies. However, there are some important problems to establish this new strategy.

The first is that the combination of imatinib and the other chemotherapies for the treatment of metastatic breast cancer have not been established and their safety and efficacy are unclear. Treatment with a combination of imatinib and cytotoxic chemotherapies have been reported in the previous several articles. Kumar et al. also reported two cases of patients with synchronous gastrointestinal stromal tumor (GIST) and colorectal adenocarcinoma (Kumar et al. 2011). They received FOLFOX chemotherapy for colon cancer while administering imatinib (400 mg/day) and did not experience unexpected toxictity from either the imatinib or chemotherapy. Uemura et al. reported secondary chronic myelogenous leukemia following postoperative S-1 (80 mg/day) therapy for advanced gastric cancer (Uemura et al. 2010). Although skin eruption, palpebral edema and appetite loss were appeared after weeks starting imatinib therapy, these symptoms were alleviated when he stopped taking imatinib. Furthermore, the results of PhaseI and II trial of combination of imatinib and chemotherapy have been reported recently. Lin et al. showed a phaseI trial of docetaxel/estramustine/imatinib in patients with hormone-refractory prostate cancer (Lin et al. 2007). Thirteen patients with hormone-refractory metastatic prostate cancer were treated every 21 days with fixed doses of estramustine (280 mg orally 3 times a day on days 1–5), imatinib (400 mg orally daily on days 1–21) and docetaxel, in which cohorts of 3–6 patients were enrolled to receive escalating doses of docetaxel on day 2 from 50 to 60 to 70 mg/m^2^. On dose level 3 (docetaxel 70 mg/m^2^ and imatinib 400 mg daily), grade 3 elevations of prothrombin time in two patients. Although the protocol was amended to include an intermediate dose level (docetaxel 60 mg/m^2^ and imatinib 300 mg daily), there were five unacceptable toxicities (2 cerebrovascular accidents, one myocardial infarction, one mesenteric ischemia, and 1 deep venous thrombosis) in 13 patients, and two of those toxicities resulted in death. Another phaseIdose-escalation study of imatinib mesylate plus capecitabine in advanced solid tumor was reported (Dugan et al. 2010). In their study, 24 patients with advanced solid tumors were treated with capecitabine twice daily on days 1–14 and imatinib mesylate once daily on a 21-day cycle. Six patients who were treated with capecitabine at 1000 mg/m^2^ and imatinib mesylate 300 mg, had unacceptable toxicity due to grade 2 intolerable hand–foot syndrome and/or grade 2 and grade 3 diarrhea. These full doses of capecitabine and imatinib mesylate were not tolerable. Although Doses were subsequently reduced to capecitabine at 750 mg/m^2^ and imatinib mesylate at 300 mg, adverse events greater than or equal to grade 3 included anemia, diarrhea, dysuria, hypophosphatemia and vertigo. There is a result of multi-institutional phase 2 study of imatinib mesylate and gemcitabine for first-line treatment of advanced pancreatic cancer (Moss et al. 2012). Gemcitabine was given at 1200 mg/m^2^ on days 3 and 10. Imatinib (400 mg) was taken orally on days 1 to 5 and 8 to 12 of a 21-day cycle. Forty-four patients were enrolled and the median number of cycles completed was three. Common adverse effects included neutropenia, nausea, anemia, and fatigue. 50 % of patients had grade 3 or higher neutropenia and 17 % had grade 3 or higher thrombocytopenia. The significant nonhematologic toxicity related to treatment were grade 3 or higher dehydration in 9 %, grade 3 or higher rash in 9 %, grade 3 or higher fatigue in 6 %, grade 3 or higher nausea in 6 %, and grade 3 or higher renal failure in 2 %. Clinically significant grade 1 and 2 toxicities included edema in 4 % and rash in 6 %. Overall, half of the patients required dose reduction.

The other problem is that the patient with PTTM generally have not been able to tolerate chemotherapy at symptom onset because of poor physical condition. Therefore, the combination therapy of imatinib and chemotherapy might not be tolerable for the patients with PTTM. It is necessary to conduct a well-designed clinical trial to evaluate the use of chemotherapies combined with imatinib for PTTM.

## Conclusion

Imatinib, which alleviated pulmonary hypertension, could be a new strategy for pulmonary tumor thrombotic microangiopathy in patient with metastatic breast cancer.

## Abbreviations

PTTM: pulmonary tumor thrombotic microangiopathy
PDGF: platelet-derived growth factor
PDGFR: platelet-derived growth factor receptor
GIST: gastrointestinal stromal tumor
